# A lentiviral toolkit to monitor airway epithelial cell differentiation using bioluminescence

**DOI:** 10.1152/ajplung.00047.2024

**Published:** 2024-08-13

**Authors:** Jessica C. Orr, Asma Laali, Pascal F. Durrenberger, Kyren A. Lazarus, Marie-Belle El Mdawar, Sam M. Janes, Robert E. Hynds

**Affiliations:** ^1^Lungs for Living Research Centre, UCL Respiratory, University College London, London, United Kingdom; ^2^Epithelial Cell Biology in ENT Research (EpiCENTR) Group, Developmental Biology and Cancer Department, Great Ormond Street UCL Institute of Child Health, University College London, London, United Kingdom; ^3^UCL Cancer Institute, University College London, London, United Kingdom

**Keywords:** airway epithelium, basal cells, lentiviral transduction, organoids, primary cell culture

## Abstract

Basal cells are adult stem cells in the airway epithelium and regenerate differentiated cell populations, including the mucosecretory and ciliated cells that enact mucociliary clearance. Human basal cells can proliferate and produce differentiated epithelium in vitro. However, studies of airway epithelial differentiation mostly rely on immunohistochemical or immunofluorescence-based staining approaches, meaning that a dynamic approach is lacking, and quantitative data are limited. Here, we use a lentiviral reporter gene approach to transduce primary human basal cells with bioluminescence reporter constructs to monitor airway epithelial differentiation longitudinally. We generated three constructs driven by promoter sequences from the *TP63*, *MUC5AC*, and *FOXJ1* genes to quantitatively assess basal cell, mucosecretory cell, and ciliated cell abundance, respectively. We validated these constructs by tracking differentiation of basal cells in air-liquid interface and organoid (“bronchosphere”) cultures. Transduced cells also responded appropriately to stimulation with interleukin 13 (IL-13; to increase mucosecretory differentiation and mucus production) and IL-6 (to increase ciliated cell differentiation). These constructs represent a new tool for monitoring airway epithelial cell differentiation in primary epithelial and/or induced pluripotent stem cell (iPSC)-derived cell cultures.

**NEW & NOTEWORTHY** Orr et al. generated and validated new lentiviral vectors to monitor the differentiation of airway basal cells, goblet cells, or multiciliated cells using bioluminescence.

## Graphical abstract

**Figure:**
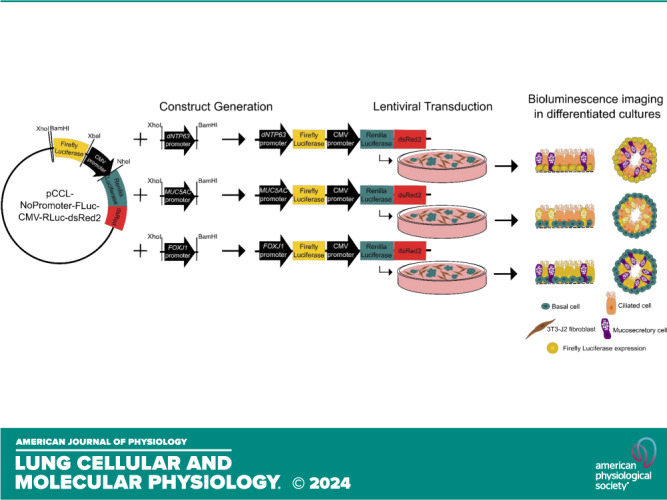

## INTRODUCTION

Airway epithelial cells play a critical role in maintaining respiratory function by forming a protective barrier against inhaled pathogens, toxins, and environmental insults. Basal stem cells, mucosecretory cells, and ciliated cells are the most abundant cell types of the airway epithelium (1). Basal cells serve as adult stem cells and maintain tissue homeostasis by repairing damage to the epithelium (2). Mucosecretory cells and ciliated cells each enact aspects of the mucociliary escalator, which acts to trap inhaled particles and remove them from the respiratory tract. Mucosecretory cells produce and secrete airway mucus, whereas ciliated cells produce motile force to move the mucus layer proximally, in doing so removing trapped matter from the respiratory system (3).

Understanding the normal development, homeostasis, and repair of the airway epithelium is crucial to understand disease states, to develop new respiratory medicines, and in the pursuit of airway tissue engineering approaches. Primary airway epithelial cell culture can achieve long-term propagation of patient-derived basal cells and protocols enable their differentiation to tissue-specific cell types (4, 5). However, studies of airway epithelial cell differentiation typically rely on either immunohistochemical and immunofluorescence staining, which are limited in their ability to provide dynamic and quantitative data, or indirect measures, such as monitoring transepithelial electrical resistance (6). The introduction of reporter genes, for example, fluorescent proteins or luciferase enzymes, can enable quantitative readout of biological activities. This is commonly achieved by transfection of plasmids, but reporter cell lines can be created by transduction of cells with lentiviral reporter constructs. Lentiviruses transduce proliferating and nonproliferating cell types and integrate the construct into the genome for stable expression. Bioluminescence imaging captures light produced by the oxidation of substrates by luciferase enzymes. The most commonly used are firefly luciferase (from the North American firefly, *Photinus pyralis*), which uses d-luciferin as its substrate and releases yellow-green light, and *Renilla* luciferase (from the sea pansy, *Renilla reniformis*), whose substrate is coelenterazine and releases predominantly blue light (7).

Tumor protein p63 (TP63) is a transcription factor that is expressed by basal cells in stratified squamous epithelia and the pseudostratified airway epithelium. Transactivating and N-terminally truncated (ΔNp63) TP63 isoforms are generated from two promoter sequences, generating at least 11 distinct isoforms (8). Airway basal cells predominantly express the ΔNp63α isoform (9). Consistent with its role in maintaining epithelial stem cells in other tissues, the knockdown of *TP63* inhibits proliferation and differentiation and promotes basal cell senescence (10). Expression of *TP63* is restricted to a subset of undifferentiated airway basal cells and reduces upon commitment to differentiation (11–14). The mucin-5AC (*MUC5AC*) gene encodes a secreted, polymeric mucin, and transcripts are restricted to airway goblet cells in the human tracheal and bronchial epithelium (15). Tobacco smoking induces *MUC5AC* expression and *MUC5AC* expression is elevated in the airways of patients with chronic obstructive pulmonary disease (COPD) (16). Variants in *MUC5AC* have been associated with chronic sputum production (17), asthma (18), and pulmonary fibrosis (19). Forkhead box protein J1 (FOXJ1) is a transcription factor involved in the late stages of ciliogenesis and, in the airways, is expressed uniquely within the multiciliated cell lineage (20). As a result of the cell-type specificity of *TP63*, *MUC5AC*, and *FOXJ1* expression, we reasoned that these would represent suitable promoter sequences to drive luciferase reporter gene expression in lentiviral reporter constructs that read out the abundance of basal cells, mucosecretory, and multiciliated cells, respectively.

## MATERIALS AND METHODS

### Luciferase Gene Reporter Constructs

To generate an editable promoter-reporter construct vector (pCLL-NoPromoter-FLuc-CMV-RLuc-dsRed2; Addgene #215329), the firefly luciferase-CMV promoter-*Renilla* luciferase-dsRed2 sequence was subcloned from the pDR5-fluc-CMV-RlucDsRed2 vector [gift from Dr. Khalid Shah (21)] using primer pairs targeting the relevant promoter sequence (Table 1), which contained SalI and XhoI sites. The DNA fragment underwent restriction enzyme digest (New England Biolabs) to generate overhangs and was subcloned into the pCCL vector.

**Table 1.** Oligonucleotide primers used for construct generation

Primer	F/R	Sequence	Promoter Region, bp	Vector Generated (Addgene #)
Reporter region	F	AATGGACCTCGAGAATGGACAATGGATCCAGATCTGCGATCTAAGTAAGC	N/A	N/A
	R	AATGGAGTCGACCTACAGGAACAGGTGGTGGCG		
*dNTP63* Promoter	F	AATGGTCTCGAGGCTGTCAGTAGGTGTAGAATTTAG	1,660	215326
	R	AATGGAGGATCCCAATATGAATCTACTTAAGAAGATAACAGA		
*MUC5AC* Promoter	F	AATTGTCTCGAGACTCACTGGGACCTTTCTGT	1,059	215327
	R	AATTCAGGATCCCAGCTTCCTCCGGCCAACAC		
*FOXJ1* Promoter	F	AATTGTCTCGAGTGAGCCGAGCCGGGACTTAG	1,098	215328
	R	AATGGAGGATCCCATGTCTGCGGGGACTCTC		

Specific genomic regions upstream of the transcription start sites of *TP63*, *MUC5AC*, and *FOXJ1* (Table 1) were then subcloned into the editable promoter-reporter construct. The *dNTP63* promoter sequence was guided by experimental data: this sequence generated the higher luciferase activity of multiple potential regulatory regions in a prior study (22). The sequences used for the *MUC5AC* (23, 24) and *FOXJ1* (25, 26) promoter-reporter constructs have been annotated as the promoter region within the cited studies.

The DNA fragments were PCR-amplified with primer pairs containing XhoI and BamHI sites from human genomic DNA (Promega) and subcloned into the pCLL-NoPromoter-FLuc-CMV-RLuc-dsRed2 vector. Complete insertion of the promoter sequence was confirmed by Sanger sequencing (GENEWIZ) and whole plasmid sequencing (Full Circle Labs).

### Data Visualization

Schematics were created in Inkscape (v1.2.1). Analyses and data visualization were performed in RStudio (v2023.12.0.369) with the tidyverse [v2.0.0 (27)] packages: dplyr (v1.1.4), tidyr (v1.3.0), tibble (v3.2.1) ggplot2 (v3.4.4), ggpubr (v.0.6.0), and the colorblind-friendly color map (Viridis, v0.6.4).

Genomic location and the sequences of genes were visualized using the Gviz package [v1.46.1 (28)]. Gene information was extracted from the ENSEMBL hg38 genome using biomaRt [v2.58.0 (29, 30)] and filtered by the HUGO gene nomenclature committee symbol.

### Cell Lines

Cell lines were cultured in incubators maintained at 37°C in 5% CO_2_. HEK293Ts were cultured in DMEM with pyruvate (Gibco) supplemented with 10% FBS (Gibco) and 1 x penicillin/streptomycin (Gibco). Penicillin/streptomycin was omitted for one passage before and during transfection. The HBEC3-KT cell line (ATCC) was cultured in airway epithelial cell basal medium (ATCC) supplemented with the bronchial epithelial cell growth Kit (ATCC) or in Keratinocyte SFM (Gibco), a serum-free medium supplemented with recombinant EGF and bovine pituitary extract. The OE19 cell line (a gift from The Francis Crick Institute Cell Services STP) and the CAPAN-2 cell line (a gift from Prof. Hemant Kocher, Barts Cancer Institute, London, UK) were cultured in RPMI (Gibco) supplemented with 10% FBS and 1 x penicillin/streptomycin. Cells were passaged using 0.05% trypsin-EDTA and were centrifuged at 300 *g* for 5 min to ensure trypsin removal.

### Lentivirus Production

Viral supernatants were created by cotransfecting HEK293T cells at 70–80% confluency in a T175 with 20-µg lentiviral construct, 13-µg pCMVR8.74 (gift from Didier Trono, Addgene plasmid #22036), and 7-µg pMD2.G (gift from Didier Trono, Addgene plasmid #12259) using JetPEI (Polyplus Transfection) following the manufacturer’s protocol. Viral supernatants were collected 48 h and 72 h posttransfection and filtered through a 0.45-µm filter (Whatman). Supernatant was combined with PEGit concentrator (5X; System Biosciences) overnight at 4°C and centrifuged at 1,500 *g* for 45 min at 4°C. The supernatant was removed, and the viral pellet was resuspended in 1/10th of the original supernatant volume in DMEM with pyruvate supplemented with 25 mM HEPES (Gibco). Concentrated supernatants were stored at –80°C until use.

### Tissue Access and Ethics Statement

Ethical approval was obtained through the National Research Ethics Committee (REC reference; 18/SC/0514). Endobronchial biopsy samples were obtained from patients undergoing bronchoscopy procedures with written informed consent.

### Primary Human Airway Epithelial Cell Culture

Cells were cultured in incubators maintained at 37°C in 5% CO_2_. 3T3-J2 mouse embryonic fibroblasts (a gift from Prof. Fiona Watt, King’s College, London, UK) were expanded in DMEM with pyruvate containing 9% bovine serum and 1X penicillin/streptomycin. These cells were mitotically inactivated by treatment with 4 µg/mL mitomycin C (Sigma-Aldrich) for 3 h to produce feeder layers. Cells were trypsinized and plated at a density of 20,000 cells/cm^2^.

Epithelial cells were added the following day in primary epithelial cell culture medium, as previously described (31, 32). Endobronchial biopsies (Table 2) were moved to the laboratory in transport medium [αMEM (Gibco) supplemented with 1x penicillin/streptomycin, 10 µg/mL gentamicin (Gibco), and 250 ng/mL amphotericin B (Fisher Bioreagents)] on ice. To generate single-cell suspensions, biopsies were incubated in 16 U/mL dispase (Corning) in RPMI for 20 min at room temperature and then mechanically disrupted using sterile forceps. This was followed by incubation in 0.1% trypsin/EDTA (Sigma-Aldrich) for 30 min at 37°C. Both dispase and trypsin/EDTA incubations were neutralized with FBS. The digested tissues were filtered through a 100-µm cell strainer (Falcon). Single-cell suspensions were centrifuged at 300 *g* for 5 min and resuspended in culture medium for counting and plating.

**Table 2.** Endobronchial biopsy donor characteristics

Donor ID	Ethnicity	Age, yr	Sex	Smoking Status	Respiratory Comorbidities Recorded
1	White	73	Male	Former	No
2	Black African	61	Male	Never	Asthma
3	White	63	Male	Never	No
4	Black African	60	Male	Current	No

Primary epithelial cell culture medium consisted of DMEM with pyruvate (Gibco) and F12 (Gibco) in a 3:1 ratio with 1X penicillin/streptomycin and 5% FBS supplemented with 5 μM Y-27632 (Cambridge Bioscience), 25 ng/mL hydrocortisone (Sigma), 0.125 ng/mL EGF (Sino Biological), 5 μg/mL insulin (Sigma), 0.1 nM cholera toxin (Sigma), 250 ng/mL amphotericin B (Fisher Scientific), and 10 μg/mL gentamicin (Gibco). The differential trypsin sensitivity of 3T3-J2 cells and epithelial cells allows the removal of feeder cells on passage (32). A first round of trypsin is added for 2–3 min, which is sufficient to remove 3T3-J2 feeder cells by aspiration. A second round of trypsin is then applied, which allows collection of the epithelial cells.

Human bronchial epithelial cell (HBEC) expansion was confirmed by immunofluorescence staining for basal cell markers TP63 and KRT5. Cell cultures were regularly screened for presence of mycoplasmas by qPCR using a previously published primer set (33).

### Lentiviral Transduction

The HBEC3-KT cell line, CAPAN-2 cell line, and primary HBECs were transduced upon passaging. Concentrated virus (100 μL) was added to 150,000 cells in suspension in a total volume of 500 μL. Cells were agitated every 5 min for 30 min to keep the cells in suspension and then plated for expansion in the appropriate cell culture medium. Medium was replaced after overnight adherence of the cells. The OE19 cell line was plated at 150,000 cells per well in a six-well plate, the following day, these cells were transduced with the addition of 100-μL concentrated virus and polybrene (4 μg/mL; Santa Cruz) to the cell culture medium. Medium was replaced 7 h after the addition of the virus.

### Fluorescence-Activated Cell Sorting

Transduced cells were enriched by fluorescence-activated cell sorting (FACS) to purify the dsRed2^+^ populations. Single-cell suspensions of epithelial cells were generated by differential trypsinization to remove the more trypsin-sensitive 3T3-J2 feeder cells. Epithelial cells were filtered through a 70-µm strainer (Falcon), centrifuged at 300 *g* for 5 min, and resuspended in FACS buffer consisting of 1% FBS, 25 mM HEPES, and 1 mM EDTA in PBS. Sorting was performed on either a BD FACS Aria or a BD FACS Aria Fusion.

### Dual Luciferase Assay

The firefly and *Renilla* luciferase activities were confirmed in the transduced cell populations using a dual luciferase assay. The transduced cell lines and nontransduced controls (HBEC3-KT, OE19, and CAPAN-2 cells) were seeded at 10,000 cells per well in a 384-well plate (Thermo Fisher Scientific) and, after 24 h, luciferase activity was determined using the Dual-Glo Luciferase Assay System (Promega) according to the manufacturer’s protocol. The relative light unit was measured on an Envision II plate reader (PerkinElmer).

### Air-Liquid Interface Culture

Air-liquid interface (ALI) culture followed a previously published method (34). Transduced primary HBECs from passages 4 (Donor IDs 3 and 4), 6 (Donor ID 1), and 8 (Donor ID 2) were used. Briefly, 0.4-μm PET transwells (Falcon) in 24-well plates (Falcon) were coated for 1 h with 50 μg/mL Collagen I (Corning) in 0.02 N acetic acid. Transwells were washed twice with PBS and air-dried for 15 min. Cells were seeded at 300,000 cells/transwell in 250 μL of primary epithelial cell culture medium. Primary epithelial cell culture medium (700 μL) was added to the basolateral side of the transwell. On *day 2*, medium was aspirated from the apical side of the transwell, exposing the confluent layer of epithelial cells to air. Medium was replaced on the basolateral side with PneumaCult medium (STEMCELL Technologies), which was prepared as per the manufacturer’s instructions. Medium was replaced twice a week and the apical side of the culture was washed once a week with PBS to remove accumulated mucus. ALI cultures underwent bioluminescence imaging on *days 2, 9, 16, 23*, and *30* and were collected for RNA isolation or fixed for immunofluorescence staining at these time points. For RNA isolation, membranes were washed twice with PBS, and cells were lysed on the membrane using RLT plus buffer and processed following the manufacturer’s protocol with the addition of an extra elution step to maximize RNA concentration (RNeasy Plus Mini Kit; Qiagen). RNA was stored at –80°C before use. For immunofluorescence staining, membranes were washed once with PBS, fixed in 4% PFA for 30 min then cut from the transwell with a scalpel and embedded in HistoGel (Epredia). After 10 min at room temperature, the gels were transferred to 70% ethanol and stored at 4°C until processing of the gels for embedding was performed using standard procedures.

### Quantitative Real-Time Polymerase Chain Reaction

A NanoDrop One (Thermo Fisher Scientific) was used to quantify RNA concentration. RNA (500 ng) was converted to cDNA using qScript cDNA SuperMix (Quanta Bio) following the manufacturer’s protocol in a 20-µL reaction. cDNA samples were diluted 1 in 2 and stored at –20°C before use. Quantitative real-time polymerase chain reaction (qPCR) was performed on a QuantStudio 5 Real-Time PCR System (Applied Biosystems) with the Power SYBR Green PCR Master Mix (Applied Biosystems). Oligonucleotide primers (Table 3) were used at 200 nM and technical triplicates were tested. Relative RNA quantification was calculated by the delta Ct method using glyceraldehyde 3-phosphate dehydrogenase (*GAPDH*) and ribosomal protein S13 (*RPS13*) as housekeeping genes. Fold change was calculated by the delta-delta Ct method.

**Table 3.** Oligonucleotide primers for qPCR

Gene	Sequence (Forward/Reverse)	Product Length, bp
*RPS13*	TCGGCTTTACCCTATCGACGCAG	153
	ACGTACTTGTGCAACACCATGTGA	
*GAPDH*	AATGAAGGGGTCATTGATGG	108
	AAGGTGAAGGTCGGAGTCAA	
*dNP63*	ATCCTGGAGCCAGAAGAAAGG	188
	TGCGCGTGGTCTGTGTTATAG	
*MUC5AC*	GCACCAACGACAGGAAGGATGAG	85
	CACGTTCCAGAGCCGGACAT	
*FOXJ1*	CAACTTCTGCTACTTCCGCC CGAGGCACTTTGATGAAGC	93

### Organoid (“Bronchosphere”) Culture

Bronchosphere culture followed a previously published method (31). Transduced primary HBECs from passages 4 (Donor IDs 3 and 4), 6 (Donor ID 1), and 8 (Donor ID 2) were used. Differentiation medium consisted of 50% DMEM with pyruvate and 50% BEBM supplemented with BEGM singlequots (except amphotericin B, triiodothyronine, and retinoic acid) (Lonza). Medium was supplemented with 100 nM all-trans retinoic acid (Sigma-Aldrich) at time of use. Briefly, wells of an ultra-low attachment 96-well plate (Corning) were coated with 30 μL of 25% matrigel (Corning) in differentiation medium and returned to the incubator at 37°C for 30 min. Epithelial cells were seeded at 2,500 cells/well in 65 μL of 5% matrigel in differentiation medium containing 5 μM Y-27632.

Bronchospheres were fed with 50-μL differentiation medium on *days 3, 10*, and *17* of culture. Where bronchospheres were supplemented with cytokines, human recombinant interleukin-6 (IL-6; PeproTech) or human recombinant IL-13 (PeproTech) were included in medium additions on *days 3, 10*, and *17*, and control wells received medium containing an equivalent amount of BSA. Bronchospheres were collected on *day 24* in ice-cold PBS and centrifuged at 300 *g* for 5 min, then fixed with 4% PFA on ice for 30 min and centrifuged at 400 *g* for 5 min. Bronchospheres were washed with ice-cold PBS and transferred to a well of a V-bottomed 96-well plate (Thermo Fisher Scientific). The plate was centrifuged at 400 *g* for 5 min, the supernatant was removed, and bronchospheres were resuspended in HistoGel. After 10 min on ice, the gels were transferred to 70% ethanol and stored at 4°C until processing of the gels for embedding was performed using standard procedures.

### Bioluminescence Imaging

Bioluminescence imaging was conducted on an IVIS Spectrum (PerkinElmer). d-Luciferin (150 µg/mL) (Abcam) was added to cell cultures in DMEM with pyruvate containing 25 mM HEPES. For ALI cultures, differentiation medium was removed from the basolateral side of the transwell and replaced with DMEM with pyruvate containing luciferin and HEPES; 200 µL was also added to the apical side of the transwell. As medium removal would disturb the bronchosphere cultures, 10-µL luciferin-containing medium was spiked into the well containing differentiation medium, giving the same final concentration as for ALI cultures.

Bioluminescent and bright-field images were taken at regular intervals from 10 min after luciferin addition until after the peak of the bioluminescent signal was recorded. This was typically within 30 min. For ALI cultures, the luminescence imaging was taken with a 1 min exposure time, medium binning, and an aperture (f/stop) of 1. For bronchosphere cultures, the bioluminescence imaging was taken with a 2 min exposure time, medium binning, and an aperture (f/stop) of 1. For all cultures, bright-field images were taken with medium binning and an aperture (f/stop) of 8.

### Histology and Immunofluorescence Staining

Samples were embedded in paraffin type 6 wax (Epredia) using an embedding station (Sakura Tissue-TEK TEC) and 5-µm sections were cut on a Microm HM 325 microtome. Hematoxylin and eosin (H&E) staining was performed on sections using an automated staining system (Sakura Tissue-Tek DRS) and imaged on a Nanozoomer Digital Pathology (Hamamatsu). Histology images were collated and presented from whole slide image files using PATHOverview (35) (available on GitHub (https://github.com/EpiCENTR-Lab/PATHOverview).

For immunofluorescence staining of samples on slides, slides were dewaxed using an automated protocol, washed in PBS, and a hydrophobic ring was drawn around the sample using an ImmEdge pen (Vector Laboratories). Sections were blocked with 1% bovine serum albumin (BSA; Merck), 5% normal goat serum (NGS; Abcam), and 0.1% Triton X-100 (Sigma-Aldrich) in PBS for 1 h at room temperature. Primary antibodies (Table 4) were diluted in block buffer and applied to slides overnight at 4°C. Slides were washed twice with PBS. Secondary antibodies conjugated to species-appropriate AlexaFluor dyes were diluted 1:1,000 in 5% NGS, 0.1% Triton X-100 in PBS, and applied to slides for 3 h at room temperature in the dark. DAPI (100 ng/mL) (Sigma-Aldrich) in PBS was applied to the slides for 20 min. Slides were washed twice in PBS and a coverslip was applied manually with Immu-Mount (Thermo Fisher Scientific). Immunofluorescence images were acquired using a Leica DMi8 microscope; the same light intensity settings were used for each combination of markers tested. To automate quantification analyses, macros were created in Fiji (36) to mask and quantify the nuclei count and area of immunofluorescent staining.

**Table 4.** Primary antibodies used for immunofluorescence staining

Antibody	Catalog Number (Supplier)	Clone	Dilution Factor
TP63	Ab124762 (Abcam)	Rabbit	1:300
KRT5	905901 (Biolegend)	Chicken	1:500
MUC5AC	M5293 (Sigma)	Mouse IgG1	1:500
ACT	T6793 (Sigma)	Mouse IgG2b	1:500
FOXJ1	14–9965-82 (ThermoFisher Scientific)	Mouse IgG1	1:200

## RESULTS

### pCLL-NoPromoter-FLuc-CMV-RLuc-dsRed2: A Versatile Lentiviral Luciferase Gene Reporter Construct

**Figure 1.**
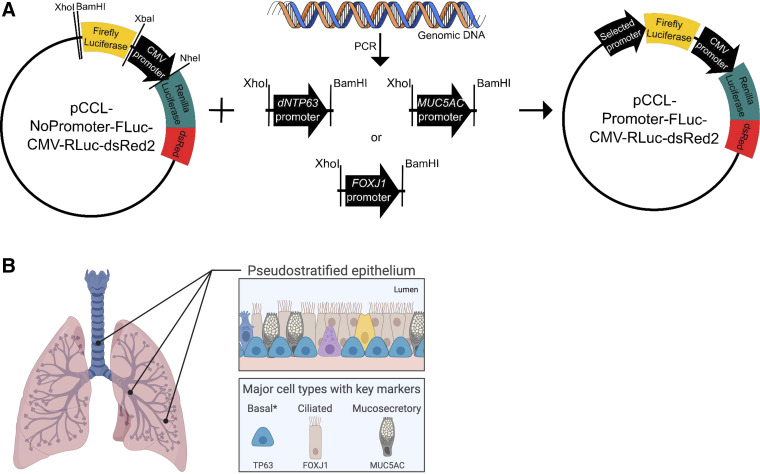
Development of a lentiviral gene reporter system for bioluminescence monitoring of airway cell type-specific gene expression. *A*: the pCCL-NoPromoter-FLuc-CMV-RLuc-dsRed2 lentiviral construct has no promoter sequence upstream of the firefly luciferase gene. *Renilla* luciferase and dsRed2 are under the control of the constitutively active CMV promoter. XhoI and BamHI restriction sites allow insertion of selected promoter sequences upstream of firefly luciferase. *B*: schematic representation of the human airway epithelium. In this study, we inserted regulatory sequences from upstream of the *dNTP63*, *MUC5AC*, and *FOXJ1* genes into independent lentiviral constructs to create reporters of basal, mucosecretory, and ciliated cell differentiation, respectively. *denotes stem cell population. [Figure created with BioRender.com.]

To generate a lentiviral reporter system suitable for monitoring airway cell type-specific gene expression, we first generated a construct in which we could insert target gene-specific promoter sequences (using the XhoI and BamHI sites) to drive firefly luciferase expression and express *Renilla* luciferase and the fluorescent protein dsRed2 from the constitutively active CMV promoter (pCLL-NoPromoter-FLuc-CMV-RLuc-dsRed2; Fig. 1*A*, *left*, henceforth “No Promoter”).

Next, we aimed to use this vector to produce cell type-specific promoter-reporter constructs for the main cell types of the pseudostratified airway epithelium (Fig. 1*B*); basal, mucosecretory, and ciliated cells. We identified sequences with literature evidence of promoter activity that were upstream of the transcription start sites of the *TP63*, *MUC5AC*, and *FOXJ1* genes, respectively (22–26). Each promoter sequence was isolated by PCR of genomic DNA with primer pairs that included the XhoI and BamHI sequences (Table 1). This enabled subcloning of each sequence into the “No Promoter” construct (Fig. 1*A*). Promoter sequence insertion was first confirmed by restriction enzyme digestion of the constructs using XhoI and BamHI, and then validated by Sanger sequencing and whole plasmid sequencing (sequences and plasmid maps are available via Addgene).

### A *dNTP63* Promoter-Reporter Construct to Monitor Basal Cell Differentiation

**Figure 2.**
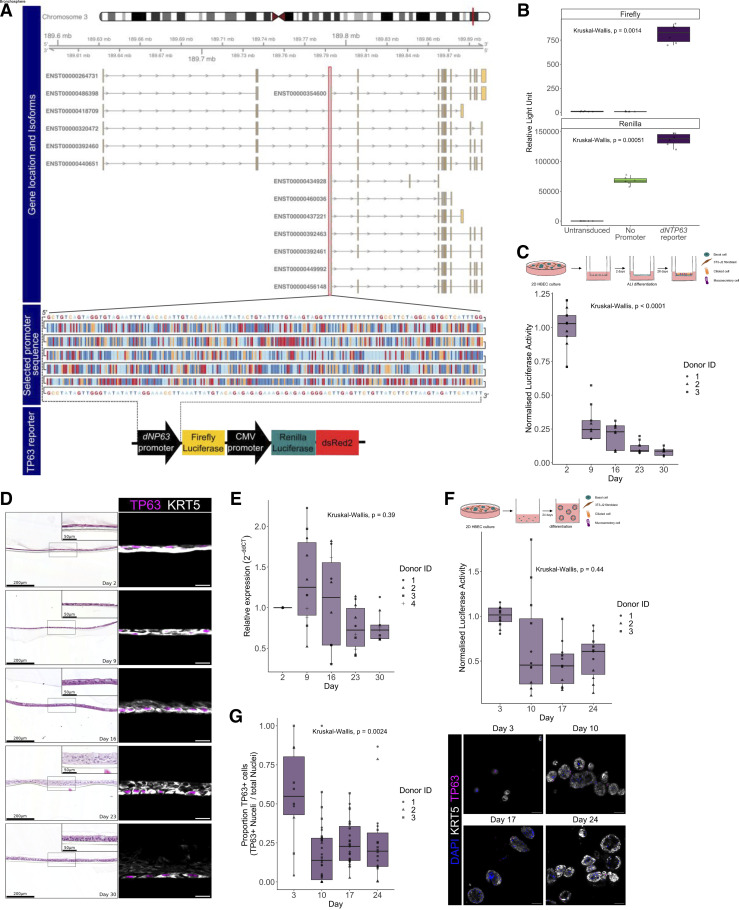
A lentiviral vector (pCLL-*dNTP63*Promoter-FLuc-CMV-RLuc-dsRed2) to monitor *TP63* expression in human airway epithelial cells. *A*: design of *dNTP63* promoter reporter vector. Genomic location of *TP63* and *TP63* isoforms (*top*). The selected *dNTP63* promoter sequence is highlighted in a red box and shown (*middle*). A schematic representation of the final *dNTP63* reporter construct is shown (*bottom*). *B*: quantification of firefly and *Renilla* bioluminescence (relative light units) in HBEC3-KT cells transduced with either the *dNTP63* reporter or the No Promoter reporter. Data shown with transduced cells from a single transduction, each data point is a single well reading. *C*: quantification of *dNTP63* promoter-driven firefly luciferase bioluminescence over time in air-liquid interface (ALI) cultures using primary human bronchial epithelial cells (HBECs) transduced with the *dNTP63* reporter (*n* = 9, 3 primary cell donors with triplicate wells per donor across 2 independent experiments). Data are normalized to the mean peak average radiance (p/s/cm^2^/sr) on *day 2* of the differentiation assay per donor. *D*: representative hematoxylin and eosin-stained and immunofluorescence images of ALI sections over time from dNTP63 reporter transduced primary human bronchial epithelial cells (Donor ID 3). Figure created with PATHOverview. Hematoxylin and eosin-stained images; scale bars = 200 μm, for inserts scale bars = 50 μm. Immunofluorescence images; scale bars = 20 μm. *E*: qPCR quantification of *dNTP63* transcript expression over time in all promoter reporter-transduced primary HBEC ALI cultures (*n* = 11 or 12, 4 primary cell donors; each data point is the mean of technical triplicates from a single transduction). *F*: quantification of *dNTP63* promoter-driven firefly luciferase bioluminescence over time in the bronchosphere differentiation assay using primary HBECs transduced with the *dNTP63* reporter (*n* = 12, 3 primary cell donors with quadruplicate wells per donor across 2 independent experiments). Data are normalized to the mean peak average radiance (p/s/cm^2^/sr) on *day 3* of the differentiation assay for each donor. *G*: quantification of immunofluorescence images of bronchosphere sections over time from *dNTP63* reporter-transduced primary human bronchial epithelial cells (*left*; *n* = 12–31, 3 primary cell donors; each data point is from a single image). Representative immunofluorescence images of basal cell markers keratin 5 (KRT5) and TP63 (right; Donor ID 3). Scale bars = 50 μm. Statistical comparisons were made using Kruskal-Wallis tests.

The *TP63* gene has multiple isoforms (Fig. 2*A*), and airway basal cells express the ΔNp63α isoform most abundantly. As such the *dNTP63* promoter sequence was used to generate the pCLL-dN*TP63*Promoter-FLuc-CMV-RLuc-dsRed2 (henceforth, “*dNTP63* reporter”) construct (Fig. 2*A*). To ensure that this construct faithfully reports the expression of *TP63*, we first transduced the HBEC3-KT cell line with lentivirus carrying the dN*TP63* reporter vector or the No Promoter control vector. After FACS to purify transduced cells based on dsRed2 expression, expression of both firefly and *Renilla* luciferases was detected in the cells transduced with the *dNTP63* reporter and only *Renilla* luciferase was detected in cells transduced with the No Promoter control vector (Fig. 2*B*).

Next, we transduced primary human bronchial epithelial cells (HBECs) with lentivirus carrying the dN*TP63* reporter vector. Transduction efficiency ranged from 5.2 to 41.0% (mean = 22.5%, *n* = 3 donors; Table 5). After FACS to purify transduced cells based on dsRed2 expression, we seeded the cells in air-liquid interface cultures and monitored luciferase expression during a 30 day time course after culture initiation (Fig. 2*C*). We observed that dN*TP63* reporter activity declined over time (Fig. 2*C*), as expected based on the differentiation of TP63^+^ basal cells to TP63^–^ luminal cell types in this assay (37). Following the luciferase assay, wells were collected for end-point analyses including histological staining to validate and visualize differentiation of transduced airway basal cells to a pseudostratified airway epithelium (Fig. 2*D*). Furthermore, air-liquid interface cultures were collected for qPCR analysis, which also showed a trend toward reduced *TP63* expression over time (Fig. 2*E*). During the course of 3-D bronchosphere differentiation, detection of firefly luciferase decreased as differentiation proceeded (Fig. 2*F*), and immunofluorescence staining for basal cell markers confirmed that the proportion of TP63^+^ cells decreased over time (Fig. 2*G*).

**Table 5.** Transduction efficiencies

Donor ID	Reporter	Transduction Efficiency (%)(DsRed+ of single cell population)
1	*dNTP63*	36.5
	*MUC5AC*	7.3
	*FOXJ1*	9.9
2	*dNTP63*	5.2
	*MUC5AC*	3.3
	*FOXJ1*	6.1
3	*dNTP63*	41
	*MUC5AC*	33.5
	*FOXJ1*	31
4	*MUC5AC*	11.9
	*FOXJ1*	12.1

### A *MUC5AC* Promoter-Reporter Construct to Monitor Mucosecretory Cell Differentiation

**Figure 3.**
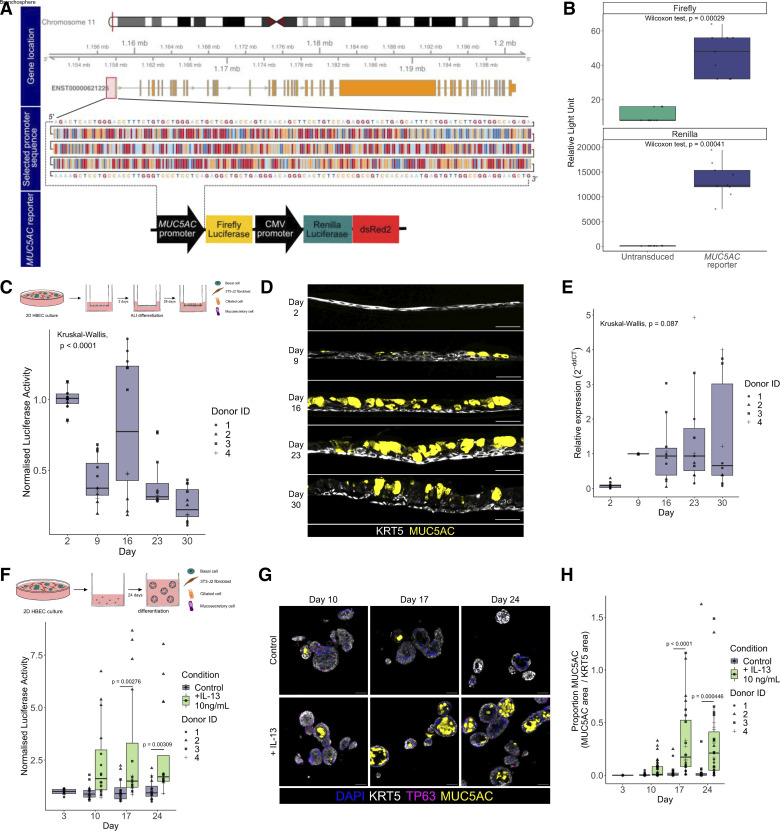
A lentiviral vector (pCCL-*MUC5AC*Promoter-FLuc-CMV-RLuc-dsRed2) to monitor *MUC5AC* expression in human airway epithelial cells. *A*: design of *MUC5AC* promoter reporter vector. Genomic location of *MUC5AC* (*top*). Selected *MUC5AC* promoter sequence is highlighted in a red box and shown (*middle*). A schematic representation of the final *MUC5AC* reporter construct is shown (*bottom*). *B*: quantification of firefly and *Renilla* bioluminescence (relative light units) in OE-19 cells transduced with the *MUC5AC* reporter. Data shown with transduced cells from a single transduction, each data point is a single well reading. A Wilcoxon nonparametric test was performed. *C*: quantification of *MUC5AC* promoter-driven firefly luciferase bioluminescence over time in air-liquid interface (ALI) cultures using primary human bronchial epithelial cells (HBECs) transduced with the *MUC5AC* reporter (*n* = 12, 4 primary cell donors with triplicate wells per donor across 2 independent experiments). Data normalized to the mean peak average radiance (p/s/cm^2^/sr) on *day 2* of the differentiation assay for each donor. A Kruskal–Wallis test was performed. *D*: immunofluorescence staining of ALI cultures derived from HBECs transduced with the *MUC5AC* promoter reporter [keratin 5 (KRT5), white; MUC5AC, yellow]; representative section images of time course cultures from donor 3. Scale bars = 50 μm. *E*: qPCR quantification of *MUC5AC* transcript expression over time in all promoter reporter-transduced primary HBEC ALI cultures (*n* = 6–8, 4 primary cell donors; each data point is the mean of technical triplicates from a single transduction. A Kruskal–Wallis test was performed. *F*: quantification of *MUC5AC* promoter-driven firefly luciferase bioluminescence over time in bronchospheres cultured in the presence of 10 ng/mL IL-13 or a BSA control using primary HBECs transduced with the *MUC5AC* reporter (*n* = 16, 4 primary cell donors; each data point is a single well reading across 2 independent experiments). Data are normalized to the mean peak average radiance (p/s/cm^2^/sr) on *day 3* of the differentiation assay for each donor. An ANOVA was performed, *P* (condition) <0.0001, *P* (day) = 0.0827. Significant Tukey test *P* values are reported between conditions on each day. *G*: immunofluorescence staining of bronchosphere cultures of *MUC5AC* reporter-transduced HBECs cultured in the presence of 10 ng/mL IL-13 or a BSA control [keratin 5 (KRT5), white; TP63, magenta; MUC5AC, yellow]; representative section images of time course cultures from donor 3. Scale bars = 50 μm. *H*: quantification of the immunofluorescence staining of bronchosphere cultures of *MUC5AC* reporter-transduced HBECs cultured in the presence of 10 ng/mL IL-13 or a BSA control. *n* = 12–47, 4 HBEC donors, each data point is from one image of a bronchosphere section. An ANOVA was performed, *P* (condition) < 0.0001, *P* (day) < 0.0001. Significant Tukey test *P* values are reported between conditions on each day.

To validate the pCCL-*MUC5AC*Promoter-FLuc-CMV-RLuc-dsRed2 (henceforth, “*MUC5AC* reporter”) construct (Fig. 3*A*), we transduced OE-19 cells, an esophageal adenocarcinoma cell line that was identified as expressing *MUC5AC* in the Human Protein Atlas cell line database (https://www.proteinatlas.org/). Activity of both firefly and *Renilla* luciferase activity was detected in the transduced cell line (Fig. 3*B*), so we proceeded to transduce primary HBECs. Transduction efficiency ranged from 3.3 to 33.5% (mean = 11.0%, *n* = 4 donors; Table 5). Following FACS purification of transduced cells and differentiation of cells in air-liquid interface cultures, we observed a decrease in luciferase expression at multiple post-airlift time points (Fig. 3*C*). In contrast, in immunofluorescence staining of sections (Fig. 3*D*) and qPCR experiments (Fig. 3*E*) from air-liquid interface cultures, we observed the expected increase in MUC5AC and *MUC5AC* expression, respectively. In 3-D bronchospheres derived from transduced basal cells, *MUC5AC* reporter expression also did not change (*P* = 0.841, two-way ANOVA). However, when we introduced the cytokine IL-13 to bronchosphere cultures to increase mucosecretory cell differentiation and expression of MUC5AC (38, 39), the luciferase signal was significantly increased [*P* (Condition) = 0.0000012, two-way ANOVA] (Fig. 3*F*). Assessment of MUC5AC protein levels in bronchospheres by immunofluorescence revealed low abundance in standard differentiation conditions, but greatly increased abundance in IL-13-treated cultures at *days 17* and *24* (Fig. 3, *G* and *H*), potentially explaining the lack of induction of the firefly luciferase reporter over time in standard differentiation conditions.

### A *FOXJ1* Promoter-Reporter Construct to Monitor Multiciliated Cell Differentiation

**Figure 4.**
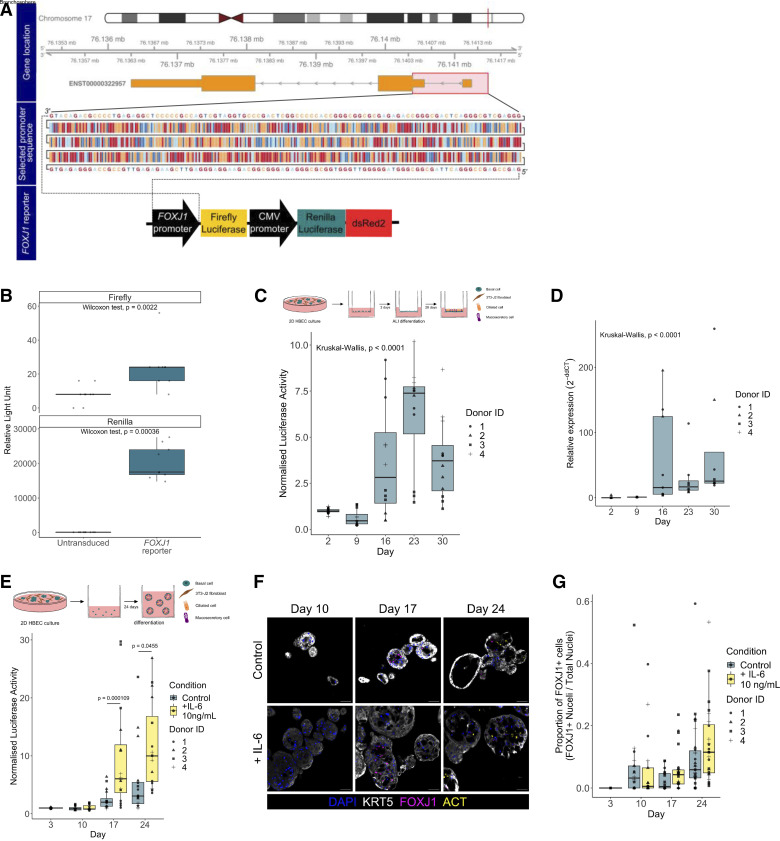
A lentiviral vector (pCLL-FOXJ1Promoter-FLuc-CMV-RLuc-dsRed2) to monitor *FOXJ1* expression in human airway epithelial cells. *A*: design of *FOXJ1* promoter reporter vector. Genomic location of *FOXJ1* (*top*). Selected *FOXJ1* promoter sequence is highlighted in a red box and shown (*middle*). A schematic representation of the final *FOXJ1* reporter construct is shown (*bottom*). *B*: quantification of firefly and *Renilla* bioluminescence (relative light units) in CAPAN-2 cells transduced with the *FOXJ1* reporter. Data shown with transduced cells from a single transduction, each data point is a single well reading. A Wilcoxon nonparametric test was performed. *C*: quantification of *FOXJ1* promoter-driven firefly luciferase bioluminescence over time in air-liquid interface (ALI) cultures using primary human bronchial epithelial cells (HBECs) transduced with the *FOXJ1* reporter (*n* = 12, 4 primary cell donors with triplicate wells per donor across 2 independent experiments). Data normalized to the mean peak average radiance (p/s/cm^2^/sr) on *day 2* of differentiation for each donor. *D*: qPCR quantification of *FOXJ1* transcript expression over time in all promoter reporter-transduced primary HBEC ALI cultures (*n* = 7 or 8, 4 primary cell donors; each data point is the mean of technical triplicates from a single transduction). *E*: quantification of *FOXJ1* promoter-driven firefly luciferase bioluminescence over time in bronchospheres cultured in the presence of 10 ng/mL IL-6 or a BSA control using primary HBECs transduced with the *FOXJ1* reporter (*n* = 16, 4 primary cell donors; each data point is a single well reading across 2 independent experiments). Data are normalized to the mean peak average radiance (p/s/cm^2^/sr) on *day 3* of the differentiation assay for each donor. An ANOVA was performed, *P* (condition) < 0.0001, *P* (day) < 0.0001. Significant Tukey test *P* values are reported between conditions on each day. *F*: immunofluorescence staining of bronchosphere cultures of *FOXJ1* reporter-transduced HBECs cultured in the presence of 10 ng/mL IL-6 or a BSA control [keratin 5 (KRT5), white; FOXJ1, magenta; ACT, yellow]; representative section images of time course cultures from donor 3. Scale bars = 50 μm. *G*: quantification of the immunofluorescence staining of bronchosphere cultures of *FOXJ1* reporter-transduced HBECs cultured in the presence of 10 ng/mL IL-6 or a BSA control. *n* = 12–31, 4 HBEC donors, each data point is from a single image of bronchosphere sections. An ANOVA was performed, *P* (condition) = 0.095, *P* (day) = 0.00052.

To validate that the pCLL-*FOXJ1*Promoter-FLuc-CMV-RLuc-dsRed2 (henceforth, “*FOXJ1* reporter”) construct (Fig. 4*A*), we transduced CAPAN-2 cells, a pancreatic ductal adenocarcinoma cell line that was identified as expressing *FOXJ1* in the Human Protein Atlas cell line database (https://www.proteinatlas.org/). Firefly luciferase activity was detected following transduction and FACS was performed based on dsRed2 expression (Fig. 4*B*). In primary cell cultures, we observed lentiviral transduction efficiencies of 6.1–31.0% (mean = 12.8%, *n* = 4 donors; Table 5). Following FACS purification of transduced cells, we observed increased *FOXJ1* reporter luciferase expression at *days 16, 23*, and *30* compared with *days 2* and *9* of air-liquid interface culture (Fig. 4*C*), consistent with the emergence of FOXJ1^+^ multiciliated cells in air-liquid interface cultures after *day 12* post-airlift (11). We validated increased *FOXJ1* transcript abundance in our cultures using qPCR (Fig. 4*D*). In 3-D bronchospheres derived from transduced basal cell cultures, we also saw increased bioluminescence at *days 17* and *24*, indicating a detectable increase in *FOXJ1* expression during the differentiation period (*P* = 0.0142, two-way ANOVA; Fig. 4*E*). Addition of IL-6 to bronchosphere cultures, which is known to promote ciliated cell differentiation in vitro (38), led to significantly increased firefly luciferase signal at *days 17* and *24* of culture compared with control cultures (Fig. 4*E*). Quantification of immunofluorescence staining for FOXJ1 showed trends toward both increased ciliated cell numbers during bronchosphere differentiation and increased ciliated cell abundance following addition of IL-6 (Fig. 4, *F* and *G*).

## DISCUSSION

To overcome some of the limitations of existing approaches for studying airway epithelial cell differentiation, and to enable real-time monitoring of the differentiation process, we developed lentiviral gene reporter constructs for basal cells, mucosecretory cells, and multiciliated cells. We developed a construct in which a cell type-specific promoter sequence drives expression of firefly luciferase and the CMV promoter drives expression of both *Renilla* luciferase and the red fluorescent protein dsRed2. dsRed2 allows facile FACS isolation of a pure population of transduced cells, while firefly luciferase can be normalized to *Renilla* luciferase to achieve a per cell quantification of promoter activity. Several primary cell cultures were transduced without seeing detrimental effects of constitutive dsRed2 expression on cell proliferation or differentiation potential over multiple passages.

We validated that promoter sequences from *TP63*, *MUC5AC*, and *FOXJ1* correlated with the abundance of relevant cell populations in primary air-liquid interface (ALI) and 3-D bronchosphere cultures. *dNTP63* promoter activity declines in ALI cultures, reflecting the reduction in expression of *dNTP63* transcripts per cell during the emergence of *TP63*^–^-differentiated epithelial cells. Furthermore, in the bronchosphere assay, the decline in *dNTP63* promoter activity over time is consistent with the transition from single airway basal cells to 3-D structures containing both TP63^ + ^KRT5^+^ basal cells at the periphery and TP63^–^-differentiated cells on the luminal surface.

We observed a relative reduction in *MUC5AC* promoter activity in ALI cultures from *day 2* to *day 9*, which was in contrast to qPCR and immunofluorescence data suggesting increases at the RNA and protein levels during differentiation. There is evidence that submerged HBECs upregulate the expression of several secretory and mucin genes, including *MUC5AC*, in the culture conditions used here compared with cultures in BEGM (32). This may explain the higher luciferase activity seen at *day 2* than later in the differentiation process. However, the lack of increased luciferase activity in both ALI and bronchosphere cultures, despite increased mRNA and protein abundance, might suggest regulation of *MUC5AC* transcript abundance by posttranscriptional mechanisms, for example, by affecting transcript stability. Despite this, the addition of interleukin 13 (IL-13), a cytokine that is known to induce mucosecretory differentiation (38, 39), to *MUC5AC* promoter reporter cells in the bronchosphere assay, resulted in increased luciferase activity on *days 17* and *24*. This mirrored the increase of MUC5AC protein observed by immunofluorescence, confirming that this lentiviral construct could be used in applications to identify modulators of airway mucosecretory differentiation. The use of gene editing to investigate the gene at the endogenous locus using fusion proteins or target gene-IRES-luciferase constructs may represent an improvement on the current method and potentially circumvent the discrepancies observed with the *MUC5AC* reporter. However, this would be technically challenging as edited, cloned, and genotyped cells would have a limited lifespan in existing culture conditions.

The expected increase in *FOXJ1* promoter activity as the differentiation assays progressed was observed by bioluminescence imaging. These results reflected the increase in *FOXJ1* transcripts in ALI culture and the emergence of FOXJ1^+^ nuclei in the bronchosphere assay. The increase of ciliated cell abundance by addition of IL-6 (40) to the bronchosphere cultures was observed by an increase in luciferase activity on *days 17* and *24*, validating the use of this lentiviral construct to identify modulators of airway ciliated cell differentiation.

The nondestructive nature of the luciferase assay on live cell cultures enables repeated measurements to be made from the same cultures over a time course, with these cultures then available for additional end-point assays (such as TEER readings, ciliary beat frequency analyses, qPCR, and immunofluorescence). Furthermore, the lentiviral constructs reported here could be useful tools in iPSC-derived airway epithelial cell differentiation protocols to provide real-time monitoring of cell-type emergence and/or to act as a quality control measure between cultures (41, 42). Given the existence of multiple luciferases that luminesce at different wavelengths (43), in the future, it may be possible to generate a lentiviral vector that monitors multiple airway epithelial cell populations simultaneously. Moreover, the cloning strategy used here created a versatile vector (pCCL-NoPromoter-FLuc-CMV-RLuc-dsRed2) into which a promoter sequence (or candidate promoter sequence) from any gene of interest can readily be inserted. In this report, we have inserted up to 1,660 bp to generate our gene reporter constructs, resulting in a 12,190-bp lentiviral vector size but the maximal capacity of these vectors has not been tested.

Limitations of the approach described here include the lack of certainty around the optimal *MUC5AC* and *FOXJ1* promoter sequences and the restriction of our study to the three major airway epithelial cell types. The former might be addressed using our pCCL-NoPromoter-FLuc-CMV-RLuc-dsRed2 vector, which could be deployed in experiments to further optimize the selected regulatory sequence to drive firefly luciferase expression in *MUC5AC*-expressing cells. Comparative testing of multiple regions and/or lengths of putative regulatory sequences could be conducted to identify the maximal firefly luciferase activity, either in OE-19 cells or in primary airway epithelial cells. Although the selection of cell types studied here represents a majority of airway epithelial cells, future studies could also investigate minor cell populations using a similar approach. For example, promoter sequences from *FOXI1* or *POU2F3* could be used to monitor the emergence of ionocytes or tuft cells, respectively (44, 45).

The lentiviruses developed here have potential applications in compound screening in primary cell cultures from healthy and diseased patient populations. As *TP63* expression correlates with stemness in stratified squamous epithelia (46), the reporter might be used to identify modulators of epithelial stemness, for example. The *FOXJ1* reporter could see application in investigations aiming to identify modulators of multiciliated cell differentiation (47), while the *MUC5AC* reporter could be relevant in various disease contexts. Goblet cell metaplasia or hyperplasia is characterized by increased differentiation of MUC5AC^+^ mucosecretory cells. As cigarette smoke exposure induces MUC5AC expression in air-liquid interface cultures (48), screening compounds for their ability to reduce smoking-induced mucosecretory cell differentiation is feasible using this approach. Similarly, airway epithelial cells from patients with asthma and COPD have higher expression levels of MUC5AC than those from healthy individuals (49–51), so screens to identify compounds to reduce goblet cell metaplasia or mucous hypersecretion would also be feasible in disease contexts.

## DATA AVAILABILITY

All relevant data are made available with this manuscript. Plasmids for the lentiviral constructs described are available via Addgene: pCCL-NoPromoter-FLuc-CMV-RLuc-dsRed2 (Addgene #215329), pCCL-dNTP63Promoter-FLuc-CMV-RLuc-dsRed2 (Addgene #215326), pCCL-MUC5ACPromoter-FLuc-CMV-RLuc-dsRed2 (Addgene #215327), and pCCL-FOXJ1Promoter-FLuc-CMV-RLuc-dsRed2 (Addgene #215328).

## GRANTS

This work was funded by grants from the Longfonds BREATH lung regeneration consortium, the UK Regenerative Medicine Platform (UKRMP2) Engineered Cell Environment Hub [Medical Research Council (MRC); MR/R015635/1] and a Rosetrees PhD Plus Award. R.E.H. was supported by a Wellcome Trust Sir Henry Wellcome Fellowship (WT209199/Z/17/Z), a NIHR Great Ormond Street Hospital BRC Catalyst Fellowship, GOSH Charity (V4322), The Royal Society (RG\R1\241421), and the CRUK Lung Cancer Centre of Excellence (C11496/A30025). S.M.J. was supported by a CRUK programme grant (EDDCPGM\100002) and an MRC programme grant (MR/W025051/1). S.M.J. is also supported by a CRUK programme grant (EDDCPGM\100002), and an MRC Programme grant (MR/W025051/1). S.M.J. receives support from the CRUK Lung Cancer Centre of Excellence (C11496/A30025) and the CRUK City of London Centre, the Rosetrees Trust, the Roy Castle Lung Cancer foundation, the Garfield Weston Trust, and University College London Hospitals Charitable Foundation. S.M.J.’s work is supported by a Stand Up To Cancer-LUNGevity Foundation American Lung Association Lung Cancer Interception Dream Team Translational Research Grant and Johnson and Johnson (SU2C-AACR-DT23-17). Stand Up To Cancer is a division of the Entertainment Industry Foundation. Research grants are administered by the American Association for Cancer Research, the Scientific Partner of SU2C. This work was partly undertaken at UCL/UCLH and partly at UCL ICH/GOSH who received a proportion of funding from the Department of Health’s NIHR Biomedical Research Centre’s funding scheme.

## DISCLAIMERS

The views expressed are those of the authors and not necessarily those of the NHS, the NIHR or the Department of Health.

## DISCLOSURES

S.M.J. has received fees for advisory board membership from BARD1 Life Sciences. S.M.J. has received grant income from GRAIL Inc. and is an unpaid member of a GRAIL advisory board. S.M.J. has received lecture fees for academic meetings from Chiesi and AstraZeneca. None of the other authors has any conflicts of interest, financial or otherwise, to disclose.

## AUTHOR CONTRIBUTIONS

J.C.O., S.M.J., and R.E.H. conceived and designed research; J.C.O., A.L., P.F.D., K.A.L., and M.-B.E.M. performed experiments; J.C.O. analyzed data; J.C.O., S.M.J., and R.E.H. interpreted results of experiments; J.C.O. prepared figures; J.C.O. and R.E.H. drafted manuscript; J.C.O., S.M.J., and R.E.H. edited and revised manuscript; S.M.J. and R.E.H. approved final version of manuscript.
